# Evaluating the Effectiveness of Competency-Based Didactic Approach (CBDA) in Pediatric Nursing Education: A Mixed-Method Study

**DOI:** 10.1155/nrp/6613710

**Published:** 2025-05-14

**Authors:** Amal Murad, Eman Mater

**Affiliations:** ^1^Maternity and Childhood Nursing Department, Nursing College, Taibah University, Medina, Saudi Arabia; ^2^Pediatric Nursing Department, Nursing College, Cairo University, Cairo, Egypt

**Keywords:** competency, didactic approach, didactic principles, performance

## Abstract

Competence-Based Didactic Approach (CBDA) supported students in developing their ability to integrate knowledge, skills, and attitudes. The study aimed to evaluate the effectiveness of CBDA in pediatric nursing education by establishing a CBD Model (CBDM) and examining the nursing students' scores and grades. The exploratory sequential mixed methods research design was used to explore the CBDM and its effect on the students' performance through their scores. Six hundred eighteen undergraduate nursing students were involved in the study. Purpose sampling was adopted to identify the CBDA impact on the nursing students score and grades. CBDM was established in parent and child nursing course to emphasize the application of didactic principles with nursing competencies. Study findings proved the highly nursing performance of CBDA among undergraduate nursing students' scores and grade. The comparison of students' scores between traditional and CBDA groups (two and three semester system) revealed highly statistically significant improvement (*p* < 0.001) which justified the research hypotheses of CBDA implementation. The total average of student scores increased from 84.54 ± 3.21 in the traditional group to 88.29 ± 1.59 in the CBDA groups. In addition to the improvement of communication skills which increased from 3.83 ± 0.36 in traditional groups to 5.88 ± 0.22 in CBDA groups. CBDA improved students' performance by emphasizing their active participation in the learning processes through the application of didactic principles in the nursing education with clear plans that clarified teaching strategies and assessment methods. CBDM is more applicable in nursing courses and improve learning outcomes and curriculum design.

**Summary**

• Education programs faced challenges to shift a knowledge-based approach of teaching and learning to a competency-based approach that emphasized by the application of CBDA to maintain sustainability development.
• Students continuously singled out a greater need for didactic instruction, which encourages curiosity, being well-informed, clarity in asking questions, open-minded, focused on the task, being diligent in the search for relevant information, prominence of self-reflection in thinking.
• The functioning of the objectives/competences become efficient at the level of the educational dimension when the didactic principles are projected, organized, and put the activities of teaching-learning-evaluating into practice and helped teachers to support students in adding the practical value of their learning.

## 1. Introduction

Introducing the competency-Based Didactic Approach (CBDA) into the educational process had the positive effect on learning process through the students began to use in-depth learning strategies more frequently than tradition learning methods [1]. Students pointed out a greater need for didactic instruction, which encourages curiosity, being well-informed, clarity in asking questions, broad minded, focused on the task, flexible, carefully making decisions, being hard working in the search for relevant information and the significance of self-reflection in thinking [2]. Engaging nursing students in academic environments and clinical settings is a challenging issue for nursing educators. In recent years, nurse researchers have investigated various educational strategies to explore and develop the best ways to increase nursing students' academic engagement. Results of these efforts are the creation of new teaching strategies or adaptation the approach used by other disciplines. Didactic competencies enhancement the quality of teaching and learning [3, 4].

Teaching and learning shifted towards competence-based approaches. Result in, examinations as well as didactical approaches need to become competence based. Unfortunately, competence-based examinations and assessments of the efficacy of didactical approaches are hard and no generally accepted approaches for competence-oriented evaluation are available. CBDA needs to be adapted to intend learning outcomes that are related to specific competences to enable students to integrate knowledge, skills, and attitudes to achieve their personal and professional development. To fulfil the aim of the study, the following research hypothesis was formulated: The Application of CBD Model (CBDM) in pediatric nursing education will enhance pediatric nursing performance. CBDM will improve pediatric nursing students' scores and grades.

## 2. Background

CBDA builds on the concept of CBD principles to help teachers to support students in developing their ability to integrate knowledge, skills, and attitudes, and adding the practical value of their learning [1, 5]. The word didactic means the art of teaching and refers to the principles, phenomena, forms, and law of teaching [6].

The didactic principles are general norms through which predicted, organized, and put the activities of teaching-learning-evaluating into practice, so that the functioning of the objectives/competences should become powerful at the level of the educational dimension. They relate to an applicative, definite dimension of the system and process of education. The didactic principles include five principles: the conscious and active participation of students in the education process, connecting theory with practice, systematization and continuity, intuition, and feedback [1, 5–7].

Competency defined as the ability to do an activity to a recommended standard, emphasizing what people can do rather than what they know. Competency based approach is a sequence of learning experiences that follow to ensure that students attain specific skills, knowledge, and important abilities to whatever they are studying [8]. Competency-based learning is learner-focused and works naturally with independent study and with the instructor acting as a facilitator. Learners often find different individual skills more difficult than others. This learning method allows a student to learn those individual skills they find challenging at their own pace, practicing and improving as much as they like. Then move rapidly to other skills to which they are more skilled. This can be determined through prior learning assessment or formative testing [9].

Conjunctive scoring models require examinees to meet performance standards for each specified content area. Competency assessment is concerned with double classification. Students classified as competent or noncompetent based on specific demonstration. Competence considered essential checklist of specific traits in which a student must demonstrate proficiency [10].

Didactic materials are vital in the teaching learning process. Educational media classified into basic media, advanced media, and community resources. Print and visual publications include the textbooks, the notebook, video tapes, pictures, and case studies. Several factors influence the selection and use of didactic material. learner characteristics are critical issues. So, the learner's entry behaviour, learning style, motivation, anxiety, socio-economic status, previous experience, special needs, specific skills/knowledge and preferences and the political environment [8].

## 3. Methods

### 3.1. Aims

The study aimed to evaluate the effectiveness of CBDA in nursing education by establish a CBDM and examine the nursing students' performance (score and grade).

### 3.2. Study Design

The exploratory sequential mixed methods research design was used to explore the CBDM and its effect on the students' grade and score that describe their performance. The initial study is qualitative, often used to explore a phenomenon of CBDA in which there is a little understanding by the application of CBDM that describe the application of didactic principles in parent and child nursing course by selecting suitable teaching strategies and evaluation methods. Then inform a second, quantitative study for exploring the effect of CBDA on students' performance.

### 3.3. Participants and Setting

Six hundred eighteen undergraduate nursing students enrolled in the parent and childhood nursing course were involved in the study for three sequential semesters in academic year 2021 (121 students), 2022 (174 students), 2023 (196 students), and retrospective year (127 students) in nursing college. Purpose sampling was adopted to identify the CBDA effectiveness in nursing students score and grades. Inclusion criteria included male and female students, age: 20–22 years-old who studied essential nursing courses such as physical assessment, fundamental nursing and medical surgical nursing courses. A power analysis was conducted to determine sample size. Using 0.05 as the level of significance, 0.95 as the power and effect size of 0.25. The minimum required sample size obtained was 400 students. A pilot study was carried out on 10% of sample size to ensure the clarity, applicability of the tools, test feasibility of the study and estimate sample size and the time needed for data collection. The result of pilot study confirmed that the study was feasible. The sample of the pilot study was excluded from the total sampling.

### 3.4. Ethical Considerations and Data Collection

The permission was obtained from maternity and child nursing department council and faculty of nursing council (number, 144414708). Data were collected through a period of 2 years from June 2021 to June 2023 and retrospective years. Didactic plans for students and teachers were developed by researcher to organize the application of teaching process such as didactic hospital training plans, course guide, corresponding matrix between competency, objectives, teaching strategies, course specification and blueprint. It was explained for teachers and students effectively. It was applied in the parent and child nursing course for three times in three academic years. The students' performance was measured by written exams and rubrics for both theoretical and practical skills. Practical skills are performed as an exhaustive set of skills, not serve as a checklist at hospital training. Students cannot necessarily do all the things in the description because of there is no two patients are identical. Students' performance is documented as competent or not competent in the logbook, but checklists are used and applied at lab training rotation and exams. Five teachers of parent and child nursing course analyzed didactic principles and wrote important statements regarding the application methods of didactic principles through the using of course documents. Then the researchers develop a structural description regarding the correlation between competencies, mile stone, domain of competencies and the application of didactic approach through a CBDM.

### 3.5. Validity and Reliability

Content Validity of the tools was assessed by five experts in the field of education from academic staff in nursing college. Their recommendation and suggestions were taken into consideration. Reliability of the quantitative tools was assessed by Cronbach's alpha (*α* = 0.82).

### 3.6. Data Analysis

The Statistical Package for the Social Science (SPSS) version 20 was utilized for data entry, tabulation, and analysis. Descriptive statistics were computed. The one-way ANOVA test was used to compare means of the student's scores.

## 4. Results

Six hundred eighteen undergraduate nursing students enrolled in the parent and child nursing course were included in the study. The age of the students in study sample ranged from 19 to 22 years old and 82.2% students were females and 17.8% students were males. The quantitative findings proved the highly nursing performance of CBDA among nursing students' scores. There was a statistically significant difference between traditional and CBDA groups (two and three semester system) regarding to the average of the students' scores and grades. The total average of student scores increased from 84.54 ± 3.21 in the traditional group to 88.29 ± 1.59 in the CBDA groups. In addition to the final practical exam (17.03 ± 1.74, 18.71 ± 1.01 and 18.74 ± 1.15, respectively) and theoretical exam (22.89 ± 2.96, 23.35 ± 4.16 and 24.37 ± 4.97) in Tables 1 and 2.

Concerning the practical semester works among traditional approach and CBDA groups (two and three semester system) revealed the improvement of communication skills which increased from 3.83 ± 0.36 in traditional group to 5.88 ± 0.22 in CBDA groups. In addition to the improvement of the total mean score of the practical semester works (27.66 ± 1.90, 28.47 ± 1.11, 28.47 ± 0.92, respectively) and the theoretical semester works such as midterm exam (8.92 ± 2.03, 8.95 ± 1.12, and 7.85 ± 1.60, respectively) in Tables 2, 3 and 4.

**Figure 1:**
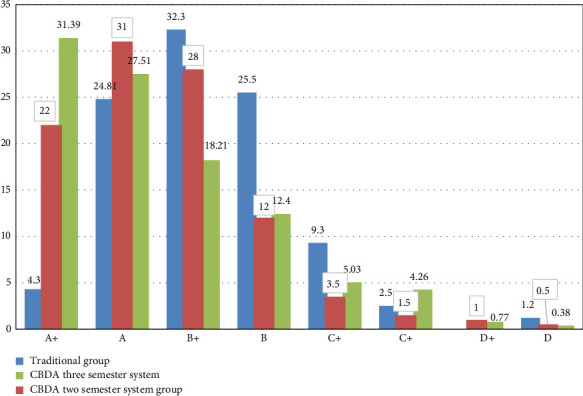
The percentage of the students' grades distributions between tradition, CBDA in two semester system and CBDA three semester system (*n* = 618). *p* < 0.000 and Chi-square: 86.14.

As displayed in Figure 1, the level of students' grades improved in CBDA groups than traditional group. The level of excellent (A+) increased from 4.3% in traditional group to 31.39% and 22% in CBDA groups (two and three semester system). Also, the level of pass (D) decreased from 0.38% in CBDA groups to 1.2% in traditional group.

Didactic principles and its conditions with the application methods in the parent and child nursing course illustrated in Table 5. The principle of the conscious and active participation of students in the education process clarified and applied through the following didactic conditions: the objectives and the competences of the didactic activity presented and explained clearly, tasks accomplished consciously and practising all the operations of thinking. Corresponding matrix between competency, objectives, and teaching strategies represented the application of this principle. Competency matrix with didactic principles represented the positive correlation between five didactic principles and competencies as shown in Table 6.

**Figure 2:**
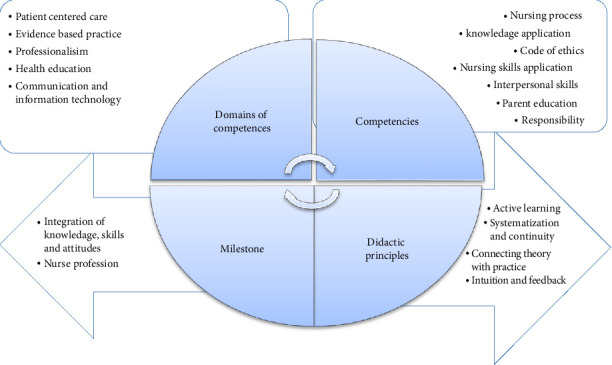
Competency-based didactic model. Mater and Murad, (2023).

CBDM illustrated the association between didactic principles, competencies, and its domain with the milestones of parent and child nursing course. The integration of knowledge, skills and attitudes and nurse profession are the milestones of paediatric nursing education that clarified with continuous process with selected competencies from the national competency framework for Bachelor of Nursing program such as nursing process, knowledge application, code of ethics, nursing skills application, interpersonal skills, parent education and responsibility. In addition to the five didactic principles that organized the application of miles stone of parent and child nursing course including the conscious and active participation of students in the education process, connecting theory with practice, systematization and continuity, intuition, and feedback in Figure 2.

## 5. Discussion

The CBDA enhanced students' performance in pediatric nursing skills through the achievement of appropriate scores in practical and theoretical competencies by the application of organized didactic plans and course guide for students and teachers that focus on the interchangeable between competencies and didactic principle to achieve pediatric nursing professionalism. So, The CBDM were developed to reflect the corresponding relation and matching between domain of competencies, competencies, principles of didactic approach and the milestone of teaching and learning that integrate knowledge, skills, and attitude.

The didactic principles reflect specific educational activities, which become essential elements for evaluating the improvement of educational process [7, 8]. Five didactic principles were applied in didactic plan of parent and child nursing course: the principle of the conscious and active participation of students in the education process, connecting theory with practice, systematization and continuity, intuition, and feedback. All didactic principles were matched with the nursing competencies; nursing process, knowledge application, code of ethics, nursing skills application, interpersonal skills, parent education and responsibility and were measured by evaluation rubrics and written exams. CBDA focused more specifically on how teachers, learners and knowledge interchange with each other. Didactic principles, when use effectively, can improve students' skills and reflect the advanced teaching and learning strategies [7, 11].

Murray [12] and Moonaghi et al. [13] stated that it is essential to provide a set of strategies for maintaining and enhancing the academic participation of nursing students. Ghasemi et al. [3] added that strategies promote nursing students' engagement. Well-defined teaching principles help to decide what, how and what content to choose, what materials and techniques to use. The formation of nursing competencies should be associated with the appropriate didactic principles for teaching [14]. The application of the didactic framework across the different programs and courses would certainly help to support faculty in the delivery of sustainability competencies [15].

Westin et al. [16] mentioned that didactic strategies helped students to learn nursing skills, solve problems and develop reflective and critical thinking and practice that supported the application of the broad knowledge on nursing and the professional role of nurses. Atashzadeh-Shoorideh et al. [17] stated that the implementation of the clinical nursing competence model and appropriate planning for achievement of nursing competencies could ensure the achievement of clinical competency by nursing students. Vargas-Hernandez and Vargas-Gonzalez [18] added that the didactic strategies help to improve the professional training. Didactic competencies in pedagogical attributes enhanced the quality of teaching and learning at the university.

The principle of the conscious and active participation of students in the education process focused on the objectives and the competences of the didactic activity should be presented and explained clearly [5–7]. It is applied through the development of a didactic hospital training plan and course guide. A hospital training didactic plan enhanced the application of learning as a goal-directed process; learners defined their own learning objectives and organized their own learning process, so learning is established in a hospital field training. It improved the students' performance of practical and theoretical part.

Vargas-Hernandez and Vargas-Gonzalez [18] reported that the didactic strategies give the students freedom and confidence, they can find their own answers and develop their knowledge, both in the classroom and in field training. The current study results showed that there was a significant difference between Traditional and CBDA groups regarding to the mean score of the final practical and theoretical exams and the total practical and theoretical semester works (*p* value: 0.000 and 0.001, respectively).

As regards to the principle of connecting theory with practice reminds of the idea that everything that is acquired from a theoretical point of view can be involved as a value at a practical level. The integration of theory and practice has been identified to be a challenge in nursing education. The assurance of the connection between theory and practice becomes possible if there are cognitive transfers taking place by the putting into value of the information to a subject. Didactic strategies are needed to provide support a reflective process that strengthens the integration between the student's lifeworld and theoretical and practical knowledge [1, 7, 16, 19].

The application of information value into practice specifically through the case study, nursing care plan and the application of skills that discussed with students. The current study results showed that there was a significant difference between Traditional and CBDA groups regarding to the mean score of the theoretical semester works (*p* value: 0.000) and practical semester works except case study and nursing care plan (*p* value: 0.000 and 0.062/0.782, respectively). Cruz-Barrientos et al. [20] reported that the implication of didactic plan through didactic simulator in the teaching-learning process among nursing students, improved the execution of development of care plans that is a fundamental work in nursing performance. Integrating clinical into didactic instructions enhanced the optimal student outcomes [21].

According to the principle of systematization and continuity. It is generated, in which the norms, the laws and the rules specific to the activities of teaching learning-evaluation interact to ensure the efficiency and the quality of the education process. One can conclude that the explanation and explication of the systemic character of the didactic principles suppose a “coherent,” “logical” educational organization of the contents, the strategies, the methods, and the forms of organizing the teaching-learning-evaluating activity meant to support the complexity of the educational act in general. [5, 7]. It was applied through course specification and guide that help students to organize their activities. Concerning the principle of feedback, efficient feedback offers relevant information regarding the quality of the learning-teaching act [5]. Feedback of students was measured through course evaluation tool with their suggestions for course improvement.

Shift from a knowledge-based approach of teaching and learning to a competency-based approach was applied by the application of CBDA that emphasized the active role of students in the learning processes, encouraging appropriate learning activities to foster a deep approach to learning [7, 8]. Therefore, competency-based education and training is an approach to teaching and learning more often used in learning concrete skills than abstract learning. So, the application of competency-based education through the didactic plans that based on the didactic principles enhanced the education process.

### 5.1. Limitation

Interpretation of the results should acknowledge some limitation; the application of CBDA in only one course in different academic years and inapplicability to measure reliability of the qualitative model.

## 6. Conclusion

The finding of the study concluded that the use of CBDA is effective to improve students' performance. The application of didactic principles in pediatric nursing education will help the achievement of intended learning outcomes of pediatric nursing course and will enhance the continues improvement of learning outcomes. CBDM illustrated the association between didactic principles, competencies, and its domain with the milestones of parent and child nursing course.

## 7. Recommendations

Based on the study results, the following recommendations are proposed:

1. An educational session is needed to raise awareness among teachers regarding CBDA.
2. Further studies needed to evaluate effect of CBDA on different courses in the same academic years and measures students' satisfactions.
3. Integrate CBDA in other nursing courses and curricula.

## 8. Implications in Nursing and Practice

The investigation demonstrated positive effect CBDA on the students' performance and enhanced the development of competency-based nursing program that maintained sustainability of program development.

## Figures and Tables

**Table 1:** Sociodemographic characteristics of traditional, CBDA in two semester system, CBDA in three semester system groups (*n* = 618).

Sociodemographic characteristics	Mean ± SD			X^2^	*P* value
	Traditional group (two semester system) (*n* = 248)	CBDA (three semester system) (*n* = 174)	CBDA (two semester system) (*n* = 196)		
*Gender*					
Female	200 (80.64%)	144 (82.75%)	164 (83.67%)		
Male	48 (19.53%)	30 (17.24%)	32 (16.32%)	0.3	0.632

*Age*					
19–20	90 (36.28%)	80 (45.97%)	100 (51.02%)		
< 20–21	120 (48.37%)	64 (36.78%)	60 (30.62%)	4.3	3.241
< 21–22	38 (15.35%)	30 (17.25%)	36 (18.36%)		

**Table 2:** The effect of CBDA on the students' mean scores of the final practical exam, final theoretical exam and total semester works in both practical and theoretical scores in relation to the traditional group (*n* = 618).

Students' scores	Mean ± SD			*F*	*P* value
	Traditional group (two semester system)	CBDA (two semester system)	CBDA (three semester system)		
Final practical exam	17.03 ± 1.74	18.71 ± 1.01	18.74 ± 1.15	103.01	0.001
Final theoretical exam	22.89 ± 2.96	23.35 ± 4.16	24.37 ± 4.97	12.23	0.001
Total practical semester works	27.66 ± 1.90	28.47 ± 1.11	28.47 ± 0.92	44.46	0.001
Total theoretical semester works	16.96 ± 1.65	17.76 ± 1.59	16.66 ± 2.21	15.32	0.001
Total	84.54 ± 3.21	88.29 ± 1.59	88.24 ± 1.35	12.34	0.001

**Table 3:** The effect of CBDA on the students' mean scores of the practical semester works in relation to the traditional group (*n* = 618).

Students' practical semester works scores	Mean ± SD			*F*	*P* value
	Traditional group (two semester system)	CBDA (two semester system)	CBDA (three semester system)		
Case study	3.63 ± 0.62	3.73 ± 0.35	3.76 ± 0.35	2.82	0.062
Parent education	2.90 ± 0.39	3.82 ± 0.29	3.86 ± 0.30	967.6	0.001
Checklist/logbook	5.68 ± 0.55	5.86 ± 0.28	5.89 ± 0.25	7.71	0.001
Communication and responsibility	3.83 ± 0.36	5.88 ± 0.22	5.89 ± 0.22	3633	0.001
Gestational age assessment	3.58 ± 0.43	3.79 ± 0.36	3.92 ± 0.15	35.2	0.001
Growth chart	1.80 ± 0.17	1.85 ± 0.17	1.97 ± 0.66	54.9	0.001
Nursing care plan	3.40 ± 0.45	3.37 ± 0.53	3.40 ± 0.36	0.24	0.782

**Table 4:** The effect of CBDA on the students' mean scores of the theoretical semester works in relation to the traditional group (*n* = 618).

Students' theoretical semester works scores	Mean ± SD			*F*	*P* value
	Traditional group (two semester system)	CBDA (two semester system)	CBDA (three semester system)		
Quiz	4.67 ± 0.44	4.31 ± 0.62	8.67 ± 1.04	511.42	0.000
Midterm exam	8.92 ± 2.03	8.95 ± 1.12	7.85 ± 1.60	16.04	
Self-instruction package (self-learning)	3.30 ± 2.11	4.71 ± 0.39	—	9.86	

**Table 5:** The application of didactic principles in parent and child nursing course.

Didactic principles	Conditions didactic principles	Application in parent and child nursing course
(1) The principle of the conscious and active participation of students in the education process	The objectives and the competences of the didactic activity must be presented and explained clearly	Corresponding matrix between competencies, objectives, teaching strategies/course specification
(2) The stimulation of the research activities must be encouraged so that the educated should acquire by himself/herself the capacity of independence in such an approach	Teaching strategies: Self-instruction package, case study, NCP, preparation of parent education session
(3) The school tasks must be accomplished consciously, practising the operation and processing of information, practising all the operations of thinking, also adopting critical attitudes referring to the use of learning strategies	The application of teaching strategies (learning strategies assessed by written exam and rubrics)

(2) The principle of connecting theory with practice	The new information must relate to the anterior experience of those who acquire it	The application of theoretical information into practice (case study, NCP,…etc)
	The putting into value of the informational content must emphasize the practical valences that the latter supposes	
	The cognitive transfers must have an important role in ensuring the connection of theory with practice	

(3) The principle of systematization and continuity	A (positive) continuity in the education process is obvious from a didactic point of view if there is a coherent, logical succession of the discourses initiated in the teaching-learning-evaluating activities	Course specifications and guide

(4) The principle of intuition	The correspondence between the mental image and the word is given by representations	Teaching strategies
	The intuitive didactic materials must be selected and used in accordance with the students' level of preparation	Simulation, videos, demonstration, pictures

(5) The principle of feedback	The immediate confirmation of behaviour supposes that at the beginning of the activity itself there should be an objective to reflect this course of action The making of an efficient feedback offers pertinent information regarding the quality of the learning-teaching act	- Use logbook as recoding method with feedback
		- Debriefing session after hospital training
		- Formative activities scores available for students immediately after each work
		- Course evaluation

**Table 6:** Competency matrix with didactic principles.

Competencies	Didactic principles (DP)				
	DP1	DP2	DP3	DP4	DP5
(1) Nursing process	✓	✓	✓	✓	✓
(2) Knowledge application	✓	✓	✓	✓	✓
(3) Code of ethics	✓	✓	✓	✓	✓
(4) Nursing skills application	✓	✓	✓	✓	✓
(5) Interpersonal skills and responsibility	✓	✓	✓	✓	✓
(6) Parent education	✓	✓	✓	✓	✓

## Data Availability

The data that support the findings of this study are available from the corresponding author upon reasonable request.
